# Evaluation of cumulative dose distributions from external beam radiation therapy using CT‐to‐CBCT deformable image registration (DIR) for cervical cancer patients

**DOI:** 10.1002/acm2.14538

**Published:** 2024-10-04

**Authors:** Carolyn Eckrich, Brandon Lee, Chunhao Wang, Kim Light, Junzo Chino, Anna Rodrigues, Oana Craciunescu

**Affiliations:** ^1^ Duke University Medical Center Department of Radiation Oncology Durham North Carolina USA; ^2^ MIM Software Cleveland Ohio USA

**Keywords:** CBCT, cervical cancer, deformable image registration

## Abstract

**Purpose:**

To investigate dose differences between the planning CT (pCT) and dose calculated on pre‐treatment verification CBCTs using DIR and dose summation for cervical cancer patients.

**Methods:**

Cervical cancer patients treated at our institution with 45 Gy EBRT undergo a pCT and 5 CBCTs, once every five fractions of treatment. A free‐form intensity‐based DIR in MIM was performed between the pCT and each CBCT using the “Merged CBCT” feature to generate an extended FOV‐CBCT (mCBCT). DIR‐generated bladder and rectum contours were adjusted by a physician, and dice similarity coefficients (DSC) were calculated. After deformation, the investigated doses were (1) recalculated in Eclipse using original plan parameters (ecD), and (2) deformed from planning dose (pD) using the deformation matrix in MIM (mdD). Dose summation was performed to the first week's mCBCT. Dose distributions were compared for the bladder, rectum, and PTV in terms of percent dose difference, dose volume histograms (DVHs), and gamma analysis between the calculated doses.

**Results:**

For the 20 patients, the mean DSC was 0.68 ± 0.17 for bladder and 0.79 ± 0.09 for rectum. Most patients were within 5% of pD for D2cc (19/20), Dmax (17/20), and Dmean (16/20). All patients demonstrated a percent difference > 5% for bladder V45 due to variations in bladder volume from the pCT. D90 showed fewer differences with 19/20 patients within 2% of pD. Gamma rates between pD and ecD averaged 94% for bladder and 94% for rectum, while pD and mdD exhibited slightly better performance for bladder (93%) and lower for rectum (85%).

**Conclusion:**

Using DIR with weekly CBCT images, the MIM deformed dose (mdD) was found to be in close agreement with the Eclipse calculated dose (ecD). The proposed workflow should be used on a case‐by‐case basis when the weekly CBCT shows marked difference in organs‐at‐risk from the planning CT.

## 1 INTRODUCTION

In 2023, an estimated 13 960 cases of invasive cervical cancer will be diagnosed with an estimated 4310 deaths as a result.^1^ For locally advanced cervical cancer patients, the standard treatment involves external beam radiation therapy (EBRT) combined with chemotherapy and brachytherapy.^2^ Prior to advances in technology, patients were treated with three‐dimensional (3D) conformal radiation therapy (CRT) with the goal of conforming the dose to the tumor while sparing the dose to surrounding normal tissues.^3^ However, specialized 3D CRT techniques, such as intensity‐modulated radiation therapy (IMRT) and volumetric modulated arc therapy (VMAT), have further improved treatments by reducing dose to normal tissue. IMRT and VMAT enable greater conformity and uniform dose distribution to a tumor while reducing damage to surrounding normal tissues.^4^ Patients with gynecological malignancies can be treated with either IMRT or VMAT plans, which depends on the complexity and location of the tumor. Before beginning treatment, a planning computed tomography scan (pCT) is obtained to design treatment plans. Following the NCCN guidelines, the standard prescription for cervical cancer EBRT is a total dose of 45–50 Gy in 1.8 Gy per fraction.^5^ Cervical cancer patients can also be treated with simultaneous integrated boost (SIB) to involved pelvic or para‐aortic (PA) lymph nodes with the SIB ranging from a total of 55 to 65 Gy delivered in 25 fractions. These plans are used to administer treatment over a period of 5−6 weeks, with reference to the pCT anatomy. The primary organs‐at‐risk (OAR) for cervical cancer RT include the bladder, rectum, and sigmoid/bowel.^6^ The EMBRACE II protocol provides recommended dose constraints and guidelines for target delineation.^7^

Pelvic organs inherently undergo displacement and changes in volume during treatment.^8^ As a result, the anatomy‐of‐the‐day during treatment may differ from the target volumes and OARs defined during planning, which can result in inaccuracies in dose delivery and an increased risk of side effects and organ toxicity, even when the target volume expansion is appropriately defined. Cone‐beam computed tomography (CBCT) images are routinely used for daily patient alignment verification, and daily anatomical variations can be observed on these images especially for the pelvic region where mobile and distensible organs such as the bladder and rectum vary in filling and position daily. To assess the impact of anatomic changes on the per‐fraction dose, several methods have been proposed in literature to calculate dose on CBCT images using different techniques such as rigid registration (RR), deformable image registration (DIR), density override algorithms, and deep learning methods.^9^

Kim et al. reviewed the different methods for cumulative dose assessment for both EBRT and brachytherapy.^10^ The methods reviewed included dose volume histogram (DVH) parameter addition, biologic dose summation, 3D RR, and 3D DIR. The results showed that when comparing DIR to parameter addition, there was no significant difference between the accumulated D2cc. However, the choice of DIR algorithm may affect the dose summation results. The results also noted that the use of RR caused inaccuracies due to soft‐tissue deformation, which resulted in registration errors on the DVH parameters of the target volume and OARs. There are challenges associated with static spatial dose summation when EBRT is delivered using highly modulated IMRT/VMAT to multiple targets with SIB. The pelvis dose distributions become highly conformal to multiple targets and often result in heterogenous dose. Linear dose summation assuming uniformity may not be valid. Teo et al. calculated cumulative dose summation for EBRT and brachytherapy, and concluded that DIR with dose summation more accurately represents dose to critical structures.^11^

Currently, clinical use of DIR is limited. AAPM TG‐132 is a guidance document for the “Use of image registration and fusion algorithms and techniques in radiotherapy.”^12^ The validation of DIR is a challenging task due to the absence of gold standards in clinical and nonclinical settings.^13^ While the dice similarity coefficient (DSC) and surface distance error are commonly used metrics for assessing the accuracy of DIR performance, studies have reported relatively high variations in these metrics depending on the algorithms being evaluated.^14^ Thus, further validation studies are necessary before implementing DIR for clinical use in dose accumulation of EBRT.^10^ While DIR has been used for dose summation in other applications, there is limited literature on its use in cervical cancer treated with EBRT. Existent published clinical data sets for cervical cancer patients that assess cumulative dose for EBRT using weekly CBCT are small. In this work, we conduct a geometrical assessment to evaluate the robustness of the DIR and compare the resulting dose distributions to determine the suitability of the DIR and dose summation techniques for clinical application in dose calculations using weekly CBCT images in patients with cervical cancer.

## 2 METHODS AND MATERIALS

### 2.1 Patients and imaging protocols

This study investigated patients with locally advanced cervical cancer recruited in accordance with an approved Institutional Review Board (IRB) protocol, treated between January 2009 and December 2018 using EBRT with concurrent chemotherapy, followed by a HDR brachytherapy boost. The external beam regimen was 1.8 Gy per fraction to 45 Gy. If patients were treated with SIB, they were treated with 2.2–2.6 Gy once daily to 55–65 Gy. The imaging prescription included a CT‐simulation to obtain the pCT, daily 2D‐2D kV radiographs, and weekly CBCT for pre‐treatment localization. The pCT images were acquired using a Philips Brilliance Big Bore CT (Philips Medical Systems, Eindhoven, Netherlands) or a Siemens Biograph PET/CT (Siemens Healthineers, Munich, Germany). The on‐treatment CBCT images were acquired on a Varian TrueBeam (Varian Medical Systems, Palo Alto, California, USA). The CBCT imaging parameters followed our pelvis protocol using 125 kVp/1080 mAs, half fan, and 360° gantry rotation.

The following normal tissues were contoured on the pCT: bladder, rectum, small bowel, large bowel (including the sigmoid), femoral heads, kidneys, and spinal cord. Departmental, site‐specific dose constraints were met for all plans. The target volumes included the GTV, CTV45, and PTV45. Eligible patients were treated with SIB. For this subset of patients, CTV/PTV55 and CTV/PTV65 were also defined. All plans were generated using Eclipse version 15.6 (Varian Medical Systems, Palo Alto, California, USA).

### 2.2 Deformable image registration algorithm

MIM Maestro v7.2.7 (MIM Software, Cleveland, Ohio, USA) was used for the DIR between the pCT and weekly CBCT images. MIM's VoxAlign Deformation Engine provides a suite of DIR algorithms. The deformation is first initialized using a RR that can be defined manually or automatically. The multi‐modality deformable registration was employed, which is a free‐form deformation that uses a feature similarity scoring metric by maximizing the correspondence of high‐dimensional feature descriptors computed by evaluating each image voxel in the context of its neighboring voxels.^15^ The DIR features of MIM were never commissioned in our practice, as we are not using it clinically. To assess its accuracy and limitations, we completed a TG‐132‐based commissioning.

#### 2.2.1 Commissioning the DIR algorithm with TG‐132

The American Association of Physicists (AAPM) TG‐132 – Use of image registration and fusion algorithms and techniques in radiotherapy provides guidelines and recommendations for quality assurance and quality control for image registration.^16^ This report provides digital phantom datasets to use for the evaluation of DIR. The datasets included a fiducial in the bladder, rectum, and prostate. Contours were manually drawn on the datasets, which included the bladder, prostate, rectum as well as landmark points added to the fiducials and centroids of each structure. According to TG‐132 recommendations, evaluating the accuracy of the deformation phantom requires the use of the deformation vector field (DVF); however, the ground truth DVF provided is in a proprietary binary format. Using recommendations by Latifi et al., and Pursley, the DSC and target registration error (TRE) were calculated.^17^, ^18^ Utilizing MIM's Deformable QA Analysis tool, the DSC and TRE were calculated for the bladder, prostate, rectum, and the landmark points. The data calculated was compared to Pursley's results. Pursley's results were calculated using MIM v6.7, whereas the results from our study were calculated using v7.2.7. The MIM DIR algorithm used for the commissioning step was the Normalized Image‐based, which was also used by Pursley. This algorithm remained unchanged between the different versions of MIM that were used for this comparison of results.

### 2.3 Dose accumulation workflow

**FIGURE 1:**
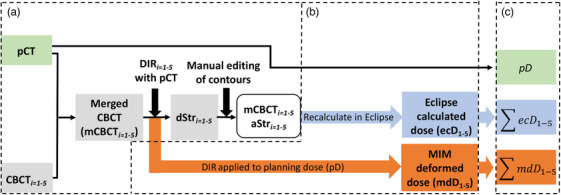
The study workflow for the (a) creation of the merged CBCT, (b) dose calculation on the merged CBCT, and (c) dose summation.

The study workflow is shown in Figure 1. Three cumulative doses will be compared and discussed in 2.5.A‐B.

- pD—clinical planning dose calculated on the pCT
- ecD—Eclipse‐calculated cumulative dose on the merged CBCT
- mdD—MIM‐deformed cumulative dose on the merged CBCT

The workflow begins with a patient's pCT and five weekly CBCT images. The pCT is merged with the CBCT to create a merged CBCT (mCBCT) with the structures from the pCT deformed (DIR*_i = 1‐5_*) to the mCBCT. The DIR‐generated contours are manually edited by a physician resulting in the adjusted structure set (aStr*_i = 1‐5_*). Further details of this step will be discussed in Section 2.4. Using the mCBCT, dose is calculated in two separate ways to quantitatively compare dose distributions using a deformation algorithm, which will be described in Section 2.5. Lastly, the dose calculated on each mCBCT is summed to generate a cumulative dose using the mCBCT for comparison against the planning dose (pD), which will be described in Section 2.6.

### 2.4 Creation of the merged CBCTs

**FIGURE 2:**
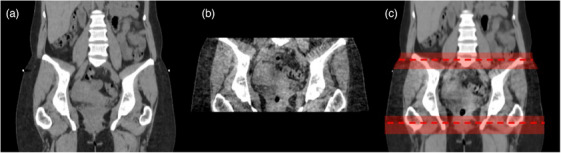
(a) pCT, (b) week 1 CBCT, and (c) mCBCT1. The edges of the original CBCT FOV are shown by the red dashed line. The rectangular regions are used for the local deformable image registration between indicate the areas used by the deformable image registration to stitch the pCT to CBCT to generate the mCBCT.

The pCT images, CBCT images, and DICOM RT‐Struct files were imported into MIM. A previous limitation with CT‐to‐CBCT DIR was the limited FOV of the CBCT. The FOV diameter of a CBCT acquired on a Varian TrueBeam is 46.5 cm and the transversal dimension from isocenter is ± 8.75 cm. The longitudinal dimension is limited to 16 cm.^19^ Using MIM's workflow “Merged CBCT,” the CBCT is deformably stitched with the pCT by deforming the anatomy of the pCT to the anatomy of the CBCT in the proximity of the CBCT FOV using a “limiting contour.”^20^ The objective of defining a limiting contour is to differentiate between the CBCT and pCT components of the merged image. To achieve this, the DIR algorithm disregards image data beyond the defined contour, focusing only on the voxels that the user specifies, which is defined as the body of the CBCT. The result is the merged CBCT (mCBCT) image stitched to the pCT. Section (a) of Figure 1 illustrates this process in the study's workflow. Figure 2 shows the pCT, the weekly CBCT, and the resulting mCBCT.

**FIGURE 3:**
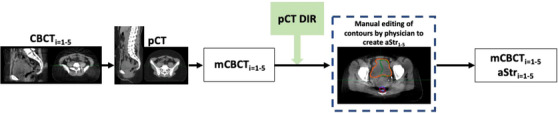
Workflow for the creation of the merged CBCT and deformed structure sets for the weekly CBCT images where the physician‐adjusted bladder = orange, DIR‐generated bladder = green, physician‐adjusted rectum = blue, and DIR‐generated rectum = brown. This process is repeated for each weekly CBCT image to generate five merged CBCT and adjusted structure sets used for this study.

A workflow designed in MIM software deformed the contours from the pCT onto the mCBCT shown in Figure 3. Due to limitations of the DIR algorithm, changes in anatomy, and poorer image quality of the CBCT, the deformed contours were adjusted and qualitatively evaluated by an expert physician.

### 2.5 Dose calculation on merged CBCT and spatial dose accumulation

Using the mCBCTs created from the pCT and the weekly CBCT, dose was calculated in two different ways for comparison to the Eclipse calculated planning dose (pD) as shown in section (b) of Figure 1.

#### 2.5.1 Creating Eclipse‐calculated cumulative dose on the merged CBCT (ecD)

The five mCBCT sets were exported from MIM to Eclipse. Five treatment plans were created: mCBCT*_i = 1‐5_*. The treatment plans were calculated with the same plan used to calculate the planned dose, pD. Dose was calculated with anisotropic analytical algorithm (AAA) (Version 15.6.03), and the leaf motion calculator used was either varian leaf motion calculator (VLMC) (Version 15.6.03) or Smart LMC (SLMC) (Version 15.6.03), matching the leaf motion calculator that was used for the clinical plan.

#### 2.5.2 Creating MIM‐deformed cumulative dose on the merged CBCT (mdD)

A workflow was created in MIM to deform the pD to the mCBCT. This process applied the DVF calculated during the image registration process to the dose distribution. The accuracy of deformable dose mapping and accumulation relies completely on the soundness of the underlying DVF created during DIR.^21^ The result is dose deformed onto each mCBCT (mdD) to be used for the next steps in the workflow.

#### 2.5.3 Spatial dose accumulation

**FIGURE 4:**
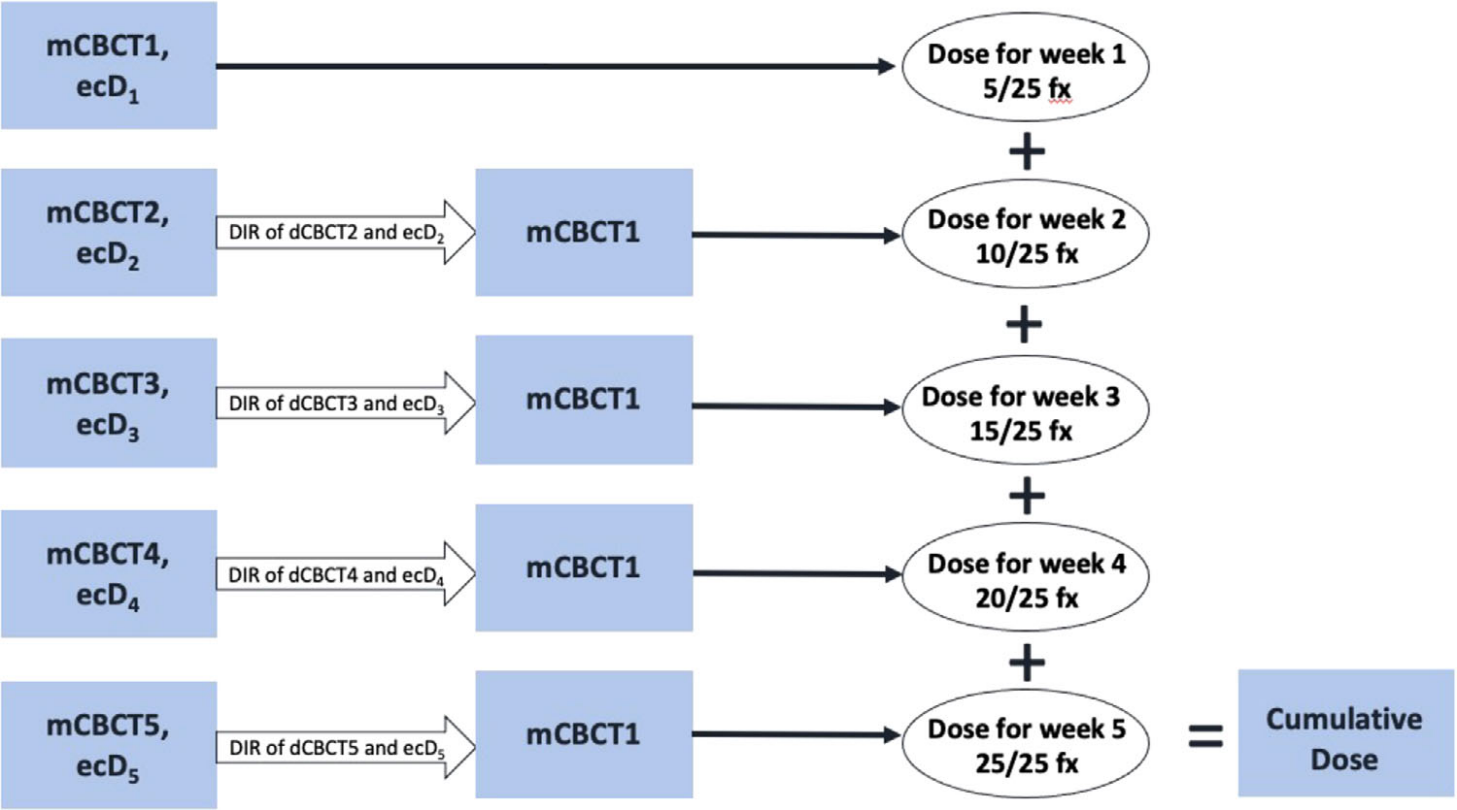
Workflow for cumulative dose to generate the cumulative dose (ΣecD_1‐5_). A corresponding accumulation workflow is used to generate (ΣmdD_1‐5_).

Spatial dose summation using DIR was used for the dose summation process with both ecD and mdD. The mCBCT1 was used as the reference image set. The subsequent mCBCTs were deformed to the reference image. The DVF is propagated onto each mCBCT to deform dose, which can then be summed between the five mCBCTs to calculate the final cumulative dose as shown in Figure 4.

### 2.6 Geometric evaluation of mCBCT to pCT DIR

A geometric assessment was conducted to evaluate the performance of the DIR in accurately mapping corresponding structures between the CT and CBCT datasets. Specifically, the output deformation field was applied to deform the features identified in the pCT and then compared them to the features manually delineated on the CBCT, which were the bladder and the rectum. The bladder and rectum were chosen to be structures that could be unequivocally identified on the CBCT and to better understand the differences present in the motility and filling of these organs throughout treatment. Due to the nature of how a PTV is created with predetermined margins, it was not necessary to deform the PTV45 onto the mCBCTs, but rather transferred from the pCT onto each mCBCT.

For each patient analyzed, the pCT structures were deformed to the mCBCTs to be used for dose calculation and dose summation in further steps. The deformed contours were reviewed and adjusted by an experienced physician. The DSC metric was used to describe the similarity between the features. The DSC quantifies the extent of overlap and shared volume between two structures.^22^ The DSC was calculated between the DIR‐generated contours and physician‐adjusted contours for each mCBCT.

### 2.7 Evaluation of dose summation calculations

The purpose of this test was to compare the pD to the cumulative doses (ecD and mdD) that were calculated on the merged CBCT images. The dose distributions were compared for the bladder (D2cc, *D*_max_, *D*_mean_, D50), rectum (D2cc, *D*_max_, *D*_mean_, D50), and PTV (D90, D98, *D*_max_, *D*_mean_) using dose‐volume histograms (DVHs). Percent differences were calculated between the pD, ecD, and mdD for the parameters listed for the bladder, rectum, and PTV. The percent difference values were categorized into three groups: ≤ ± 0%−2%, ± 2%−5%, and > ± 5%. Statistical analysis was performed with the Wilcoxon signed rank test where differences between the three doses were considered statistically significant if *p* < 0.05.

The dose distributions within the bladder and rectum for the calculated doses (ecD and mdD) and reference dose (pD) were also compared using gamma analysis (3%/2 mm, 90% pass rate, 20% threshold).^23^

## 3 RESULTS

### 3.1 Patient selection

**FIGURE 5:**
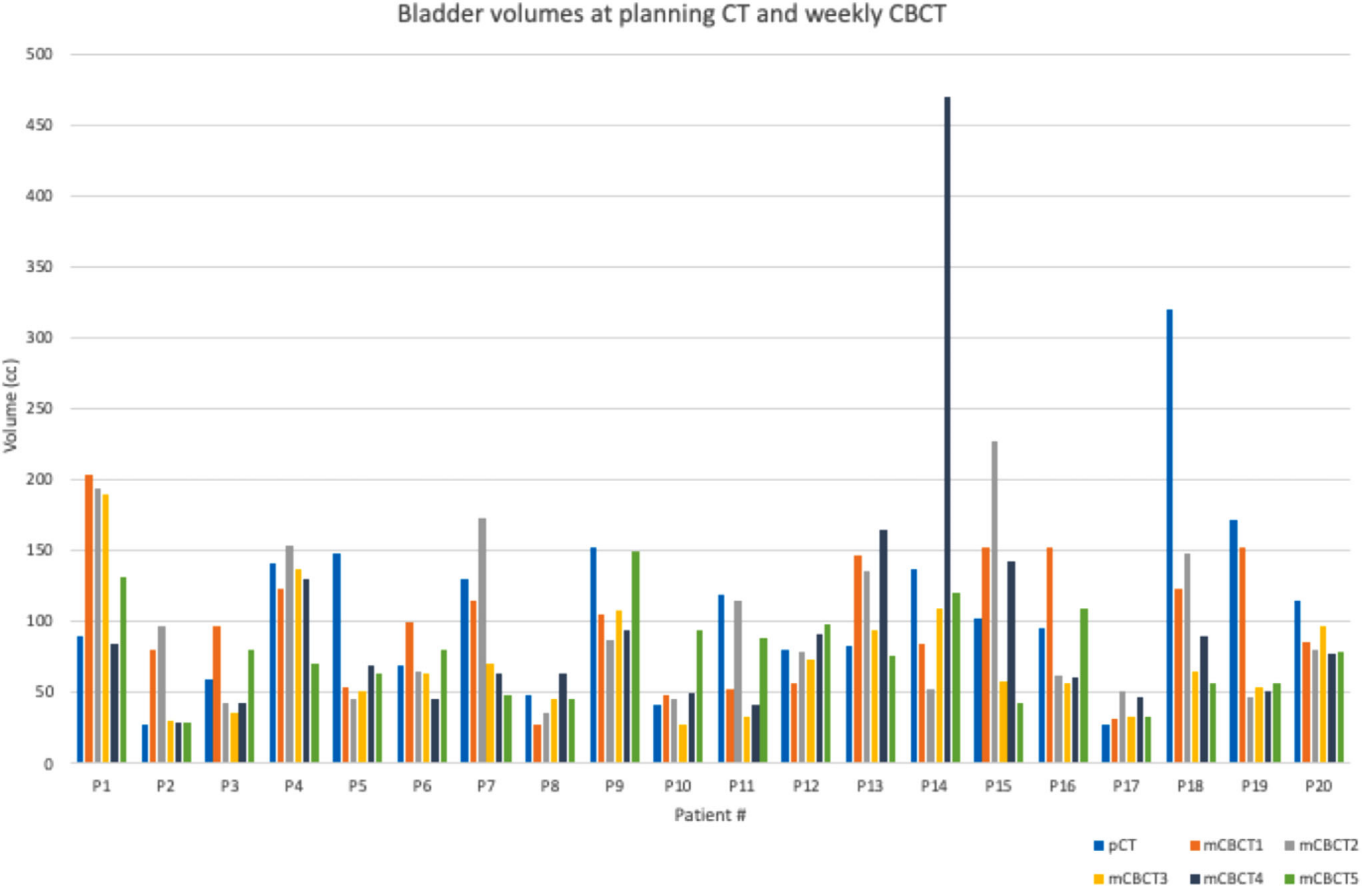
Bladder volumes at planning CT and at each weekly CBCT for each patient.

**FIGURE 6:**
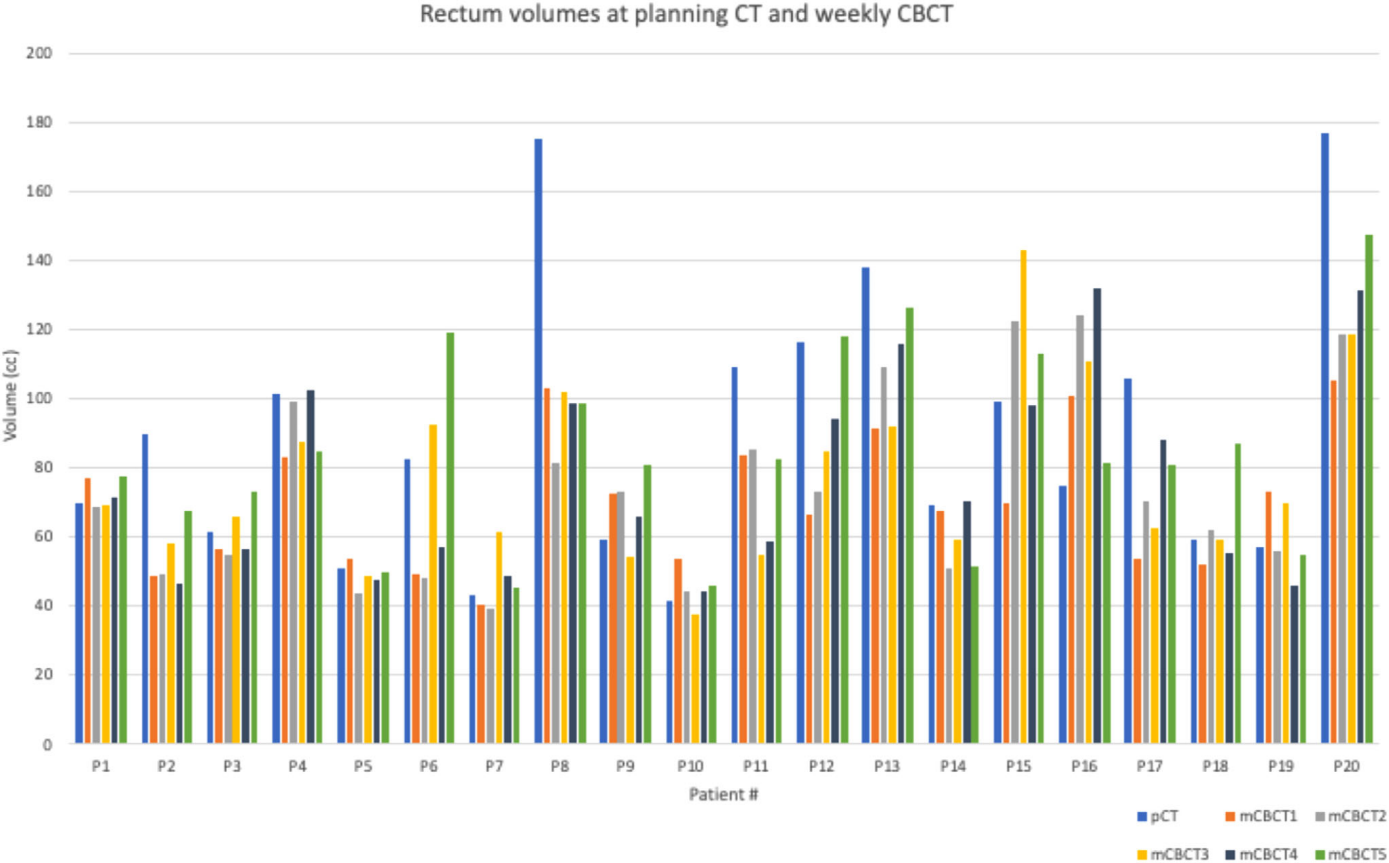
Rectum volumes at planning CT and at each weekly CBCT for each patient.

Retrospective data from 20 locally advanced cervical cancer patients treated in our clinic were used in this study. Patient eligibility was based on factors such as image quality of weekly CBCT, disease extent, and patient size. Our institution does not follow a bladder filling protocol for our gynecological (GYN) patients. This study illustrated the variation in bladder filling and positioning on an interfraction basis as shown in Table 1 and Figures 5 and 6.

**TABLE 1:** Bladder and rectum volumes are shown for each patient at time of planning CT and the percent difference from the weekly mCBCT as well as the median percent difference across the five mCBCTs.

	Bladder							Rectum						
	pCT (cc)	mCBCT1 (%)	mCBCT2 (%)	mCBCT3 (%)	mCBCT4 (%)	mCBCT5 (%)	median (%)	pCT (cc)	mCBCT1 (%)	mCBCT2 (%)	mCBCT3 (%)	mCBCT4 (%)	mCBCT5 (%)	median (%)
P1	89	227	217	212	−94	148	212	70	110	−98	−100	103	111	103
P2	28	288	349	107	102	104	107	90	−54	−55	−65	−51	−75	−55
P3	59	164	−73	−62	−72	137	−62	62	−91	−89	107	−92	119	−89
P4	141	−87	−109	−97	−92	−50	−92	101	−82	−98	−87	101	−84	−84
P5	149	−36	30	−34	−47	−42	−36	51	106	−86	−96	−93	−97	−93
P6	68	145	94	−93	−66	117	94	83	−60	−58	112	−69	144	−58
P7	131	−88	132	−54	−49	−37	−49	43	−95	−92	143	113	106	106
P8	48	−58	−76	−95	133	−96	−76	175	−59	−47	−58	−56	−56	−56
P9	152	−69	−57	−71	−62	−98	−69	59	123	124	−92	112	137	123
P10	41	117	110	−64	120	228	117	42	129	107	−91	106	111	107
P11	119	−43	−96	−28	−35	−73	−43	109	−77	−78	−50	−54	−76	−76
P12	80	−70	−99	−92	115	123	−70	116	−57	−63	−73	−81	101	−63
P13	83	176	163	113	199	−92	163	138	−66	−79	−67	−84	−92	−79
P14	136	−62	−39	−80	344	−88	−62	69	−97	−74	−86	102	−74	−74
P15	103	148	221	−56	139	−42	139	99	−70	123	144	−99	114	114
P16	96	159	−65	−59	−64	113	−59	75	135	166	147	176	109	147
P17	27	115	189	123	174	121	123	106	−51	−67	−59	−83	−76	−67
P18	320	−39	−46	−20	−28	−18	−28	59	−88	105	100	−93	147	100
P19	172	−89	−27	−31	−30	−33	−31	57	129	−98	123	−80	−96	−80
P20	114	−74	−69	−84	−68	−69	−69	177	−60	−67	−67	−74	−84	−67

### 3.2 Commissioning of DIR using TG‐132

**FIGURE 7:**
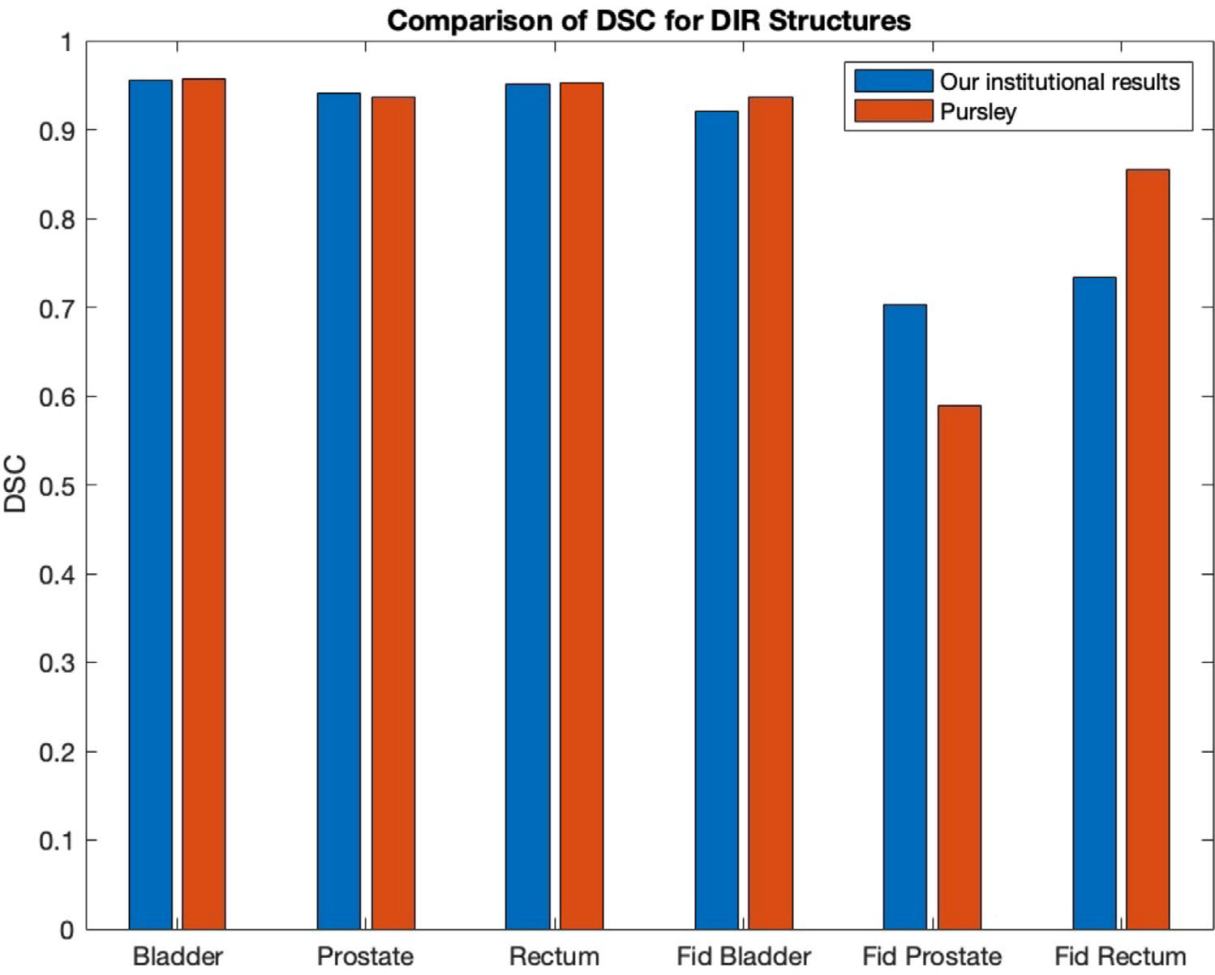
Dice similarity coefficient comparison between contoured structures on TG‐132 dataset.

The results, shown in Table 2 and Figure 7, were compared to Pursley.^18^

**TABLE 2:** Comparison of this study's and Pursley's mean DSC and TRE of the contoured structures using the TG‐132 phantoms.

	Mean DSC	Mean TRE (mm)
Our institutional results	0.867 ± 0.117	2.496 ± 2.871
Pursley^18^	0.893 ± 0.117	2.691 ± 2.515

### 3.3 Geometric evaluation of ecD and mdD

**FIGURE 8:**
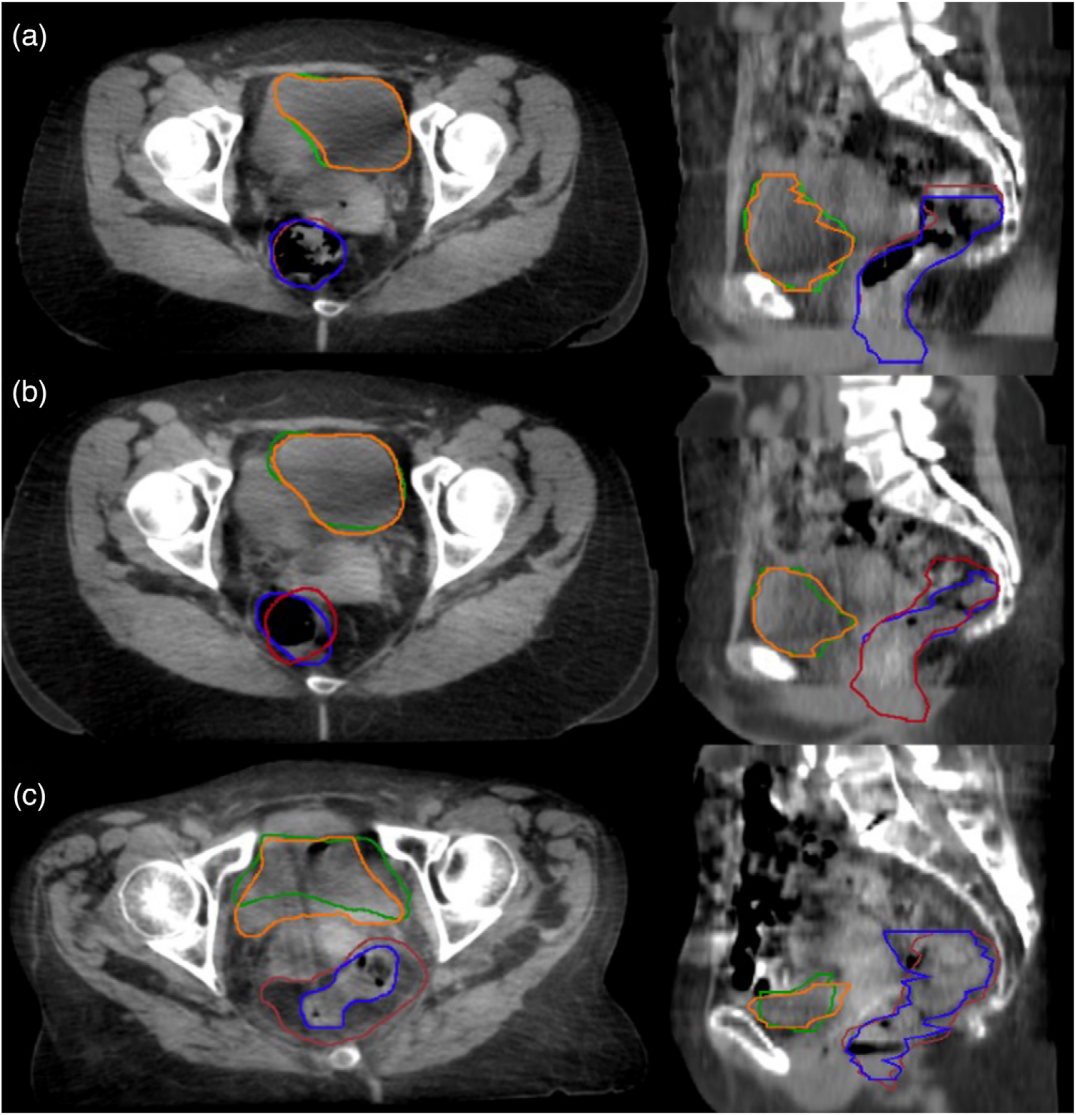
Physician‐adjusted bladder = orange, DIR‐generated bladder = green, physician‐adjusted rectum = blue, and DIR‐generated rectum = brown where (a) DSC > 0.9 (b) DSC between 0.6 and 0.8 (c) DSC < 0.6.

For the 20 patients analyzed, the bladder and rectum contours were modified on each mCBCT. Patients were assigned to categories based on the level of modifications necessary to the DIR‐generated contours according to the DSC. Figure 8 displays a representative patient for each of these defined categories.

**FIGURE 9:**
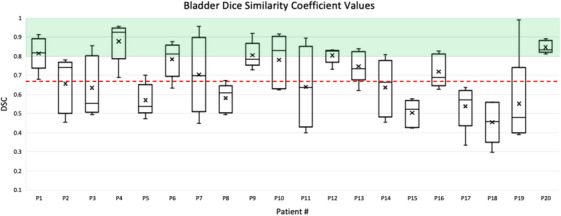
Box and whisker plot of the bladder DSC between the DIR‐generated contours and the physician‐adjusted contours for 5 mCBCTs per patient. The average DSC (0.68 ± 0.17) is shown by the red dashed line. The green shading indicates a DSC ≥ 0.8. Patients 7, 11, and 19 show a larger interquartile range. Due to large bladder filling variations between treatments, the volume of the bladder on the weekly CBCT differed from that of the pCT causing the DIR‐generated contours to match the volume of the pCT bladder more closely. The DIR‐generated bladder contours required adjustments made by a radiation oncologist. Patient 7 will be discussed further.

**FIGURE 10:**
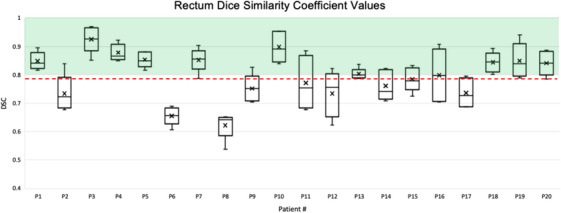
Box and whisker plot of the rectum DSC between the DIR‐generated contours and the physician‐adjusted contours for five mCBCTs per patient. The average DSC (0.79 ± 0.09) is shown by the red dashed line. The green shading indicates a DSC ≥ 0.8. Patient 6 and 8 both show lower DSC values with a mean DSC across mCBCT1‐5 of 0.66 ± 0.03 for patient 6 specifically. Variations in rectal gas and filling between the pCT and weekly CBCT contributed to adjustments made by a Radiation Oncologist to the DIR‐generated contours. Patient 8 had a pCT rectum volume of 174cc; however, the mean rectum volume between mCBCT1‐5 was 96 cc with a corresponding mean DSC of 0.62 ± 0.04. The weekly CBCT fillings were more similar to one another compared to the original volume at time of the pCT.

The average DSC was 0.68 ± 0.17 for bladder and 0.79 ± 0.09 for rectum. Appendix A presents the DSC values for each mCBCT for the 20 patients analyzed. The mean and standard deviation for each patient's DSC values for the five mCBCTs are also shown. Figures 9 and 10 show a box plot for each patient to visualize the distribution of values across each patient's mCBCT. For full DSC results per patient, refer to Table A1 and Table A2.

### 3.4 Dose evaluation of ecD and mdD

#### 3.4.1 Bladder

Regardless of the method of dose calculation on the mCBCT, 19 out of 20 patients were within 5% of the pD for the D2cc, 17 out of 20 patients for the *D*_max_, and 16 out of 20 patients for *D*_mean_ as shown in Table 3. Eleven patients were within 2% of the pD for D2cc, 12 patients for *D*_max_, and 10 patients for *D*_mean_. Patient 6 experienced the largest difference among the D2cc for the three calculated doses with the largest difference between the ecD and the pD at 15.30%. For V45, all patients had a percent difference > 5% between the mdD and pD due to the large variations in bladder volume as shown earlier in Table 1. Statistically significant differences were noted for D2cc between the pD and ecD (*p* = 0.0139) and the ecD and mdD (*p* = 0.00078). For D50, statistically significant differences were noted between the pD and ecD (*p* = 0.00152) and the ecD and mdD (*p* = 0.00008).

**TABLE 3:** Percent difference for bladder D2cc, *D*_max_, *D*_mean_, and V45.

	D2cc			*D*_max_			*D*_mean_			V45		
	ecD, pD	mdD, pD	mdD, ecD	ecD, pD	mdD, pD	mdD, ecD	ecD, pD	mdD, pD	mdD, ecD	ecD, pD	mdD, pD	mdD, ecD
P1	4.5%	0.9%	−3.6%	11.3%	3.2%	−6.6%	0.6%	8.8%	−0.7%	129.0%	123.6%	−2.4%
P2	4.0%	−0.2%	−3.9%	3.1%	−0.9%	−4.1%	4.0%	9.8%	−3.7%	189.3%	190.2%	0.3%
P3	1.1%	0.3%	−0.8%	0.7%	0.1%	−0.6%	1.1%	0.2%	−0.9%	65.9%	62.1%	−2.3%
P4	−4.6%	−2.2%	2.5%	−0.9%	−0.5%	0.5%	−0.6%	−1.2%	−0.7%	−16.3%	−21.4%	−6.2%
P5	0.1%	−0.7%	−0.6%	−1.9%	0.6%	2.6%	0.2%	−0.3%	−0.5%	−65.5%	−66.3%	−2.5%
P6	15.3%	9.3%	−5.2%	7.2%	5.9%	−1.3%	4.8%	2.7%	−2.1%	63.5%	58.6%	2.4%
P7	−0.1%	−0.6%	−0.5%	4.0%	−0.7%	−4.5%	−0.1%	−0.2%	−0.2%	−14.3%	−12.2%	2.4%
P8	0.3%	0.2%	−0.1%	−0.4%	−0.5%	−0.1%	3.2%	0.6%	−2.6%	−24.0%	−42.8%	−24.7%
P9	−0.2%	−0.7%	−0.5%	−0.6%	−1.2%	−0.7%	0.0%	−0.7%	−0.7%	−34.5%	−33.4%	1.7%
P10	−1.1%	−2.4%	−1.4%	1.2%	−0.4%	−1.5%	0.7%	−0.9%	−1.6%	17.1%	17.1%	0.0%
P11	2.4%	−2.0%	−4.3%	2.6%	−1.5%	−4.0%	2.0%	−2.4%	−4.3%	−4.8%	−81.2%	−80.3%
P12	1.3%	−0.5%	−1.8%	1.1%	−0.5%	−1.6%	6.4%	3.0%	−3.2%	−5.7%	−15.0%	−9.8%
P13	1.7%	0.1%	−1.6%	1.5%	−0.1%	−1.6%	2.8%	−0.7%	−3.4%	−116.9%	70.6%	−21.4%
P14	−0.2%	−0.4%	−0.2%	−0.2%	−0.8%	−0.6%	−13.2%	−14.5%	−1.6%	−47.9%	−52.4%	−8.7%
P15	3.9%	3.6%	−0.2%	1.4%	0.9%	−0.5%	1.6%	1.2%	−0.5%	49.6%	49.3%	−0.2%
P16	1.9%	0.1%	−1.8%	1.8%	0.0%	−1.8%	1.6%	−1.4%	−2.9%	61.9%	48.1%	−8.5%
P17	4.5%	0.9%	−3.5%	13.9%	11.4%	−2.2%	1.8%	0.8%	−1.0%	16.3%	16.3%	0.0%
P18	1.0%	−0.5%	−1.5%	2.4%	0.0%	−2.4%	3.5%	1.3%	−2.1%	−48.2%	−53.3%	−9.8%
P19	2.5%	−0.2%	−2.6%	2.4%	−0.3%	−2.7%	3.5%	−0.8%	−4.1%	1.0%	−14.3%	−15.2%
P20	0.7%	−0.4%	−1.1%	0.7%	−0.5%	−1.1%	1.0%	−0.4%	−1.4%	−21.1%	−27.7%	−8.3%

Percent differences were calculated using the planning dose as reference. Green: Indicates ≤ ± 0%−2%, yellow: ± 2%−5%, and red: > ± 5%.

#### 3.4.2 Rectum

Similarly, regardless of the dose calculation method, 18 out of 20 patients were within 5% of the pD for the D2cc. Thirteen out of 20 patients were within 5% of the pD for *D*_max_, and 11 out of 20 were within 5% of the pD for *D*_mean_ shown in Table 4. Fifteen patients were within 2% of the pD for D2cc, nine patients for *D*_max_, and six patients for *D*_mean_. Statistically significant differences between the pD and ecD (*p* = 0.0041), pD and mdD (*p* = 0.01046), and ecD and mdD (*p* = 0.00008) for D2cc.

**TABLE 4:** Percent difference for rectum D2cc, *D*_max_, *D*_mean_, and V45.

	D2cc			D_max_			D_mean_		
	ecD, pD	mdD, pD	mdD, ecD	ecD, pD	mdD, pD	mdD, ecD	ecD, pD	mdD, pD	mdD, ecD
P1	0.9%	0.2%	−0.7%	3.5%	5.1%	1.6%	1.2%	1.6%	0.4%
P2	3.1%	−0.6%	−3.6%	2.9%	−1.2%	−4.0%	−2.1%	−3.6%	−1.5%
P3	1.3%	−0.1%	−1.4%	1.4%	−0.1%	−0.1%	−2.4%	−1.7%	−1.7%
P4	0.1%	−0.7%	−0.9%	−5.4%	−3.6%	2.0%	−3.3%	−7.5%	−4.4%
P5	0.1%	−0.1%	−0.2%	−4.2%	−2.4%	1.9%	0.4%	0.1%	−0.3%
P6	0.0%	−0.4%	−0.4%	0.3%	1.3%	1.0%	2.1%	6.2%	4.1%
P7	−0.1%	−0.2%	−0.1%	−0.4%	−0.7%	−0.3%	−4.1%	−1.8%	2.4%
P8	1.1%	−0.6%	−1.7%	1.6%	−1.0%	−2.5%	−1.1%	−0.9%	0.2%
P9	1.0%	0.3%	−0.7%	0.7%	−0.5%	−1.1%	0.9%	1.8%	0.9%
P10	12.4%	0.2%	−10.9%	18.7%	3.5%	−12.9%	5.8%	0.9%	−4.6%
P11	2.4%	−0.2%	−2.5%	1.0%	−1.6%	−2.5%	−1.3%	−0.3%	1.1%
P12	1.5%	−0.5%	−2.0%	1.2%	−0.7%	−1.9%	1.6%	−4.2%	−5.7%
P13	1.8%	−0.6%	−2.3%	1.5%	−0.8%	−2.3%	−10.2%	−10.3%	−0.2%
P14	−0.3%	−0.6%	−0.3%	10.5%	11.0%	0.4%	−2.7%	−1.0%	1.8%
P15	1.3%	−1.2%	−2.4%	−3.1%	−9.2%	−6.4%	−5.5%	−8.3%	−2.9%
P16	2.4%	0.0%	−2.3%	2.2%	−0.2%	−2.4%	5.0%	3.7%	−1.2%
P17	−1.0%	−1.4%	−0.4%	−4.6%	−3.4%	1.3%	−7.4%	−4.1%	3.5%
P18	7.6%	0.9%	−6.2%	16.8%	10.1%	−5.7%	−0.1%	−1.5%	−1.3%
P19	3.9%	−0.3%	−4.0%	−5.6%	−0.3%	5.6%	3.7%	−0.5%	−4.1%
P20	−0.5%	−1.0%	−0.5%	−0.6%	−1.0%	−0.4%	−15.1%	−7.7%	8.8%

Percent differences were calculated using the planning dose as reference. Green: Indicates ≤ ± 0%−2%, yellow: ± 2%−5%, and red: > ± 5%.

#### 3.4.3 PTV45

The three doses performed similar to one another for D90 with 19 out of 20 patients within 2% of the pD. Eighteen out of 20 patients were within 5% for D98 with the largest difference for patient 8, which reached −23.00% between the mdD and pD shown in Table 5. Six patients were within 2% for D98.

**TABLE 5:** Percent difference for PTV45 D90 and D98.

	D90			D98		
	ecD, pD	mdD, pD	mdD, ecD	ecD, pD	mdD, pD	mdD, ecD
P1	0.3%	0.2%	−0.1%	−2.1%	−0.3%	1.8%
P2	−0.2%	−0.3%	−0.1%	−1.2%	−1.0%	0.1%
P3	−1.9%	−1.7%	0.2%	−5.2%	−4.0%	1.3%
P4	0.1%	−0.6%	−0.7%	−1.0%	−3.9%	−3.0%
P5	0.7%	−0.8%	−1.5%	0.1%	−2.5%	−2.6%
P6	−0.6%	−0.2%	0.4%	−2.8%	−3.0%	−0.2%
P7	−0.1%	0.1%	0.2%	−0.4%	0.1%	0.5%
P8	−1.3%	−7.1%	−5.8%	−3.5%	−23.0%	−20.2%
P9	−0.9%	−0.1%	0.9%	−4.5%	−2.1%	2.5%
P10	1.0%	0.1%	−0.9%	1.3%	−1.1%	−2.3%
P11	1.9%	−1.2%	−3.0%	0.8%	−2.6%	−3.3%
P12	0.7%	−1.3%	−1.9%	−1.4%	−4.9%	−3.5%
P13	0.7%	−0.7%	−1.4%	−0.1%	−2.6%	−2.5%
P14	−0.5%	−0.9%	−0.4%	−1.5%	−2.2%	−0.7%
P15	−0.2%	−0.5%	−0.3%	−0.4%	−2.8%	−2.4%
P16	0.7%	−0.5%	−1.2%	−0.6%	−1.5%	−1.0%
P17	0.2%	−0.2%	−0.4%	−1.2%	−1.5%	−0.3%
P18	1.3%	−0.3%	−1.6%	1.3%	−1.0%	−2.3%
P19	0.8%	0.0%	−0.8%	−0.6%	−0.5%	0.1%
P20	−0.1%	−0.6%	−0.5%	−3.6%	−3.8%	−0.2%

Percent differences calculated in same manner as Tables 3 and 4.

**TABLE 6:** Gamma passing percentages between the ecD and mdD with the pD using a 3%/2 mm pass‐fail criteria.

	Gamma for pD and ecD		Gamma for pD and mdD	
	Bladder	Rectum	Bladder	Rectum
P1	98%	99%	99%	93%
P2	100%	100%	100%	85%
P3	99%	91%	98%	96%
P4	100%	85%	100%	56%
P5	100%	100%	100%	100%
P6	95%	94%	79%	70%
P7	96%	93%	100%	99%
P8	100%	89%	62%	91%
P9	96%	98%	86%	96%
P10	98%	80%	100%	84%
P11	39%	80%	100%	88%
P12	73%	97%	100%	53%
P13	97%	92%	81%	94%
P14	96%	95%	97%	99%
P15	100%	100%	99%	77%
P16	98%	96%	83%	99%
P17	99%	99%	97%	80%
P18	89%	98%	99%	79%
P19	97%	99%	95%	96%
P20	100%	99%	84%	60%

Patients with ≥90% passing rate are highlighted in green and failing gamma pass rates (<90%) are shown in red.

Statistically significant differences were observed for D90 between the pD and mdD (*p* = 0.00054) and ecD and mdD (*p* = 0.00278). Statistically significant differences were observed for D98 between the pD and ecD (*p* = 0.00634), pD and mdD (*p* = 0.001), and ecD and mdD (*p* = 0.01596).

#### 3.4.4 DVH comparison

**FIGURE 11:**
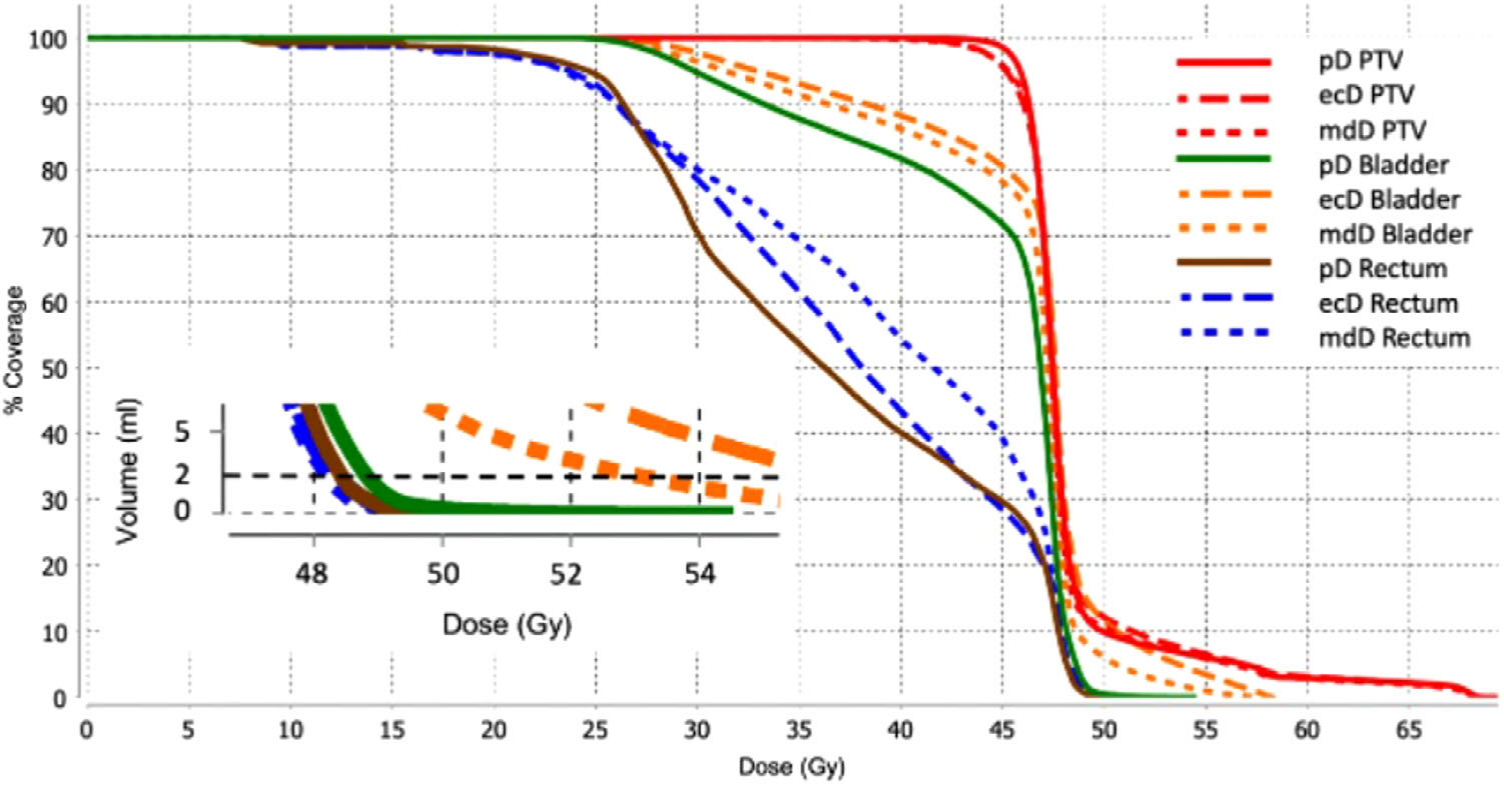
Patient example DVH showing a > 5% difference between bladder and rectum pD, ecD, and mdD. Insert shows zoomed in DVH for D2cc.

**FIGURE 12:**
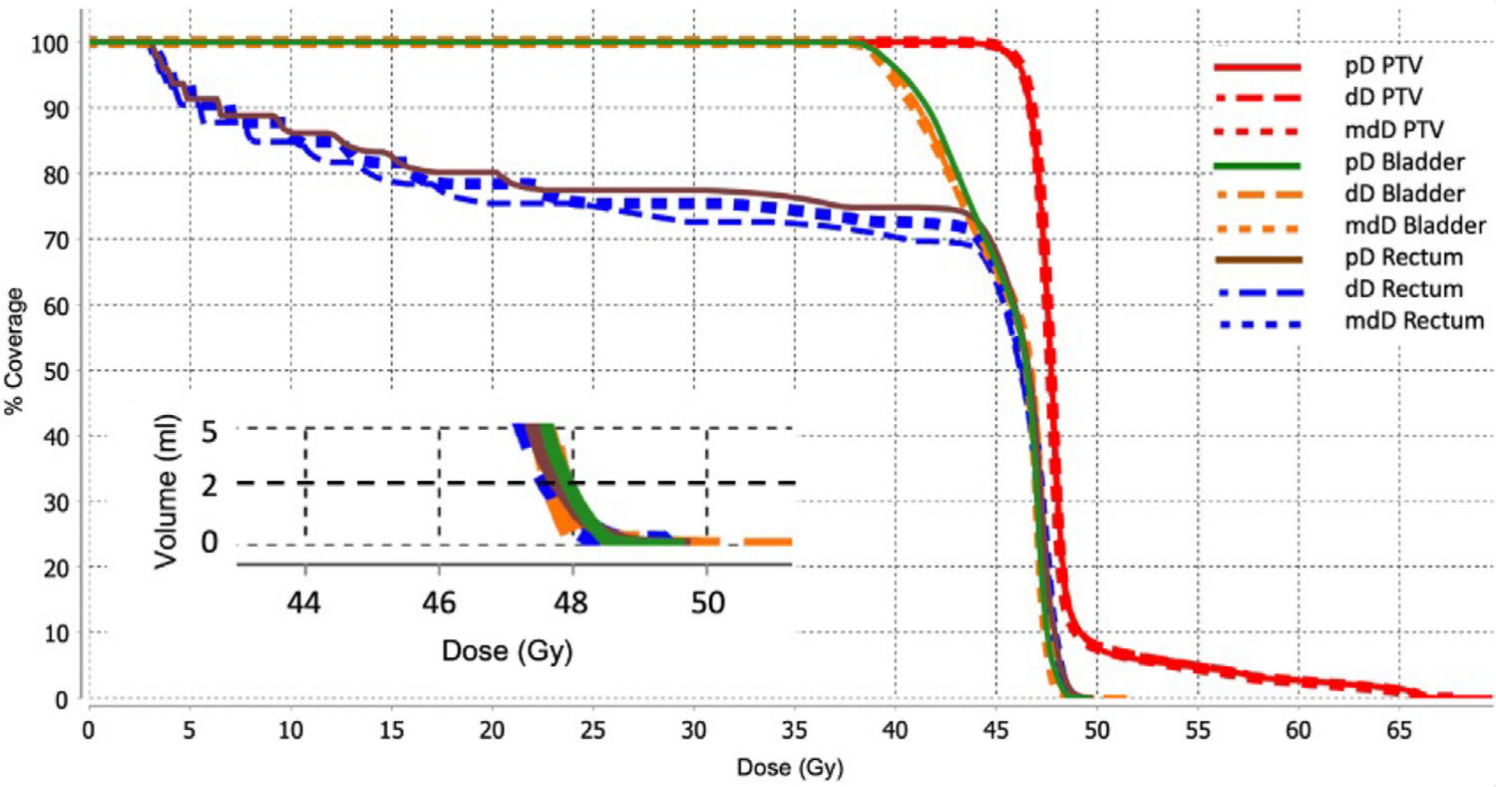
Patient example DVH showing ± 0%−5% difference between bladder and rectum pD, ecD, and mdD. Insert shows zoomed in DVH for D2cc.

Figures 11 and 12 show two separate patient DVH examples for one patient who had a > 5% difference between bladder and rectum doses and a patient with 0%−5% difference.

#### 3.4.5 Gamma analysis

**FIGURE 13:**
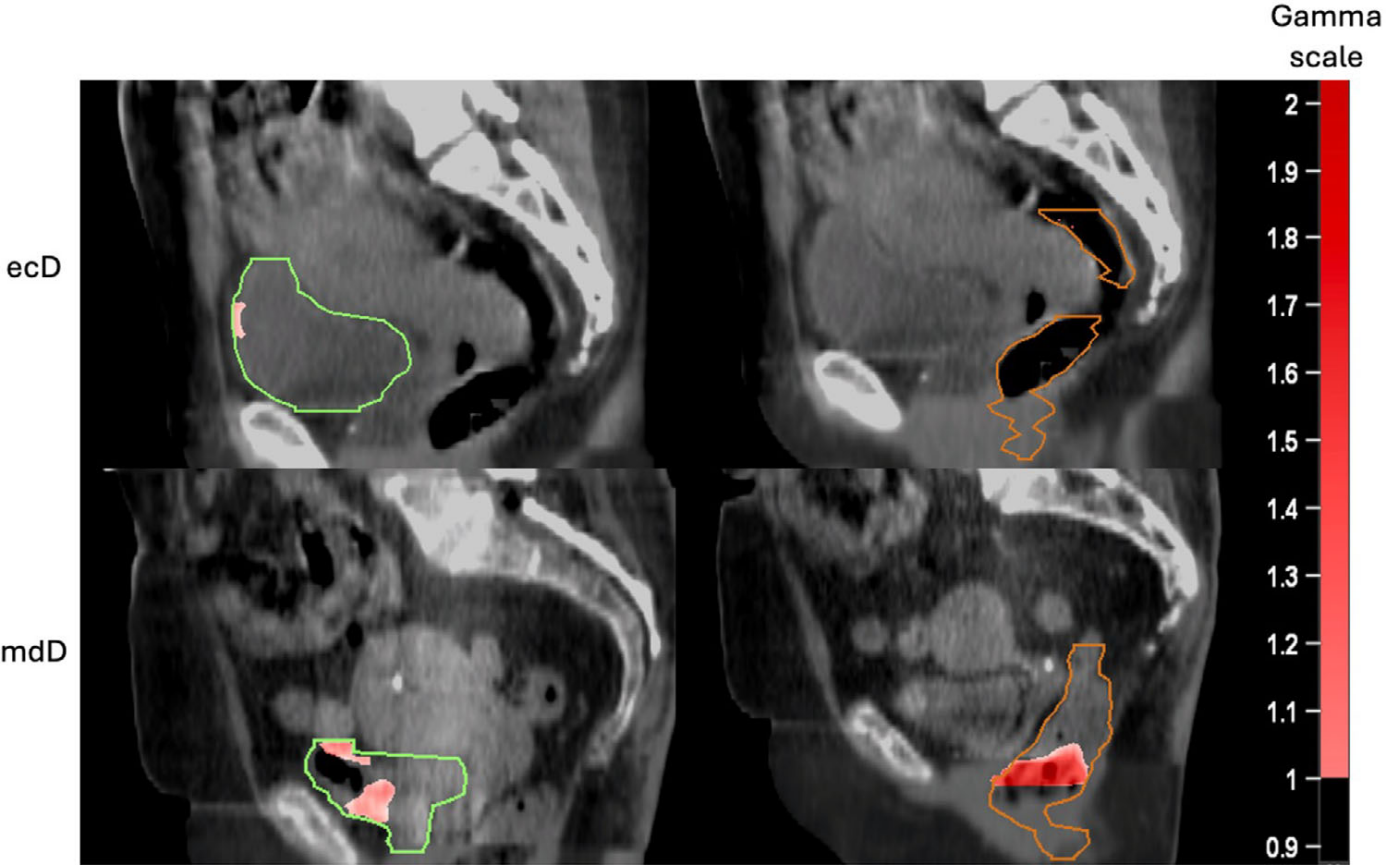
The passing gamma analysis results between pD and ecD are shown on the top row for patient 1 and the failing gamma analysis results between pD and mdD are shown on the bottom row for patient 20. The reference structure set is mCBCT1. The bladder is shown in green and rectum in brown.

The average gamma pass agreement rates between pD and ecD was 94% for bladder and 94% for rectum. The average pass rate for bladder between pD and mdD performed slightly worse with 93% but performed 9% lower for rectum with 85%. Table 6 shows the gamma pass rates for each patient. Figure 13 displays an example patient's gamma analysis results for the bladder and rectum.

## 4 DISCUSSION

This study demonstrated a reasonable agreement between the planning dose and weekly CBCT using DIR and cumulative dose in dose‐volume metrics, except in situations where CBCT image quality was poor and there were significant variations in organ filling. The mCBCT image sets were created using MIM's DIR algorithm and dose was calculated and summed.

The available literature guiding the validation of image registration software, especially DIR, is still relatively new. The challenge is multifaceted, as the determination of successful registration relies on various factors, including the algorithm, site, metrics, and clinical objectives.^17^ TG‐132 offers a practical set of simulated models and standards for validating RR; however, little guidance is provided for the evaluation of the DIR. Similar to the field in general, there is a lack of well‐characterized ground truth information when it comes to the validation of DIR. Assessing the DVFs seems to be the most thorough approach to validate the DIR; however, creating clinically relevant ground truth DVFs is challenging and demands specialized software tools.^17^ The report provides a DVF in a proprietary binary format, which led to no DVF comparisons in this study. This trend extends to most published papers evaluating commercial DIR software. The validation method used in this study involved the DSC and TRE metrics for defined contours and landmark points, which was also used in similar studies.^17^, ^18^, ^22^, ^24^

**FIGURE 14:**
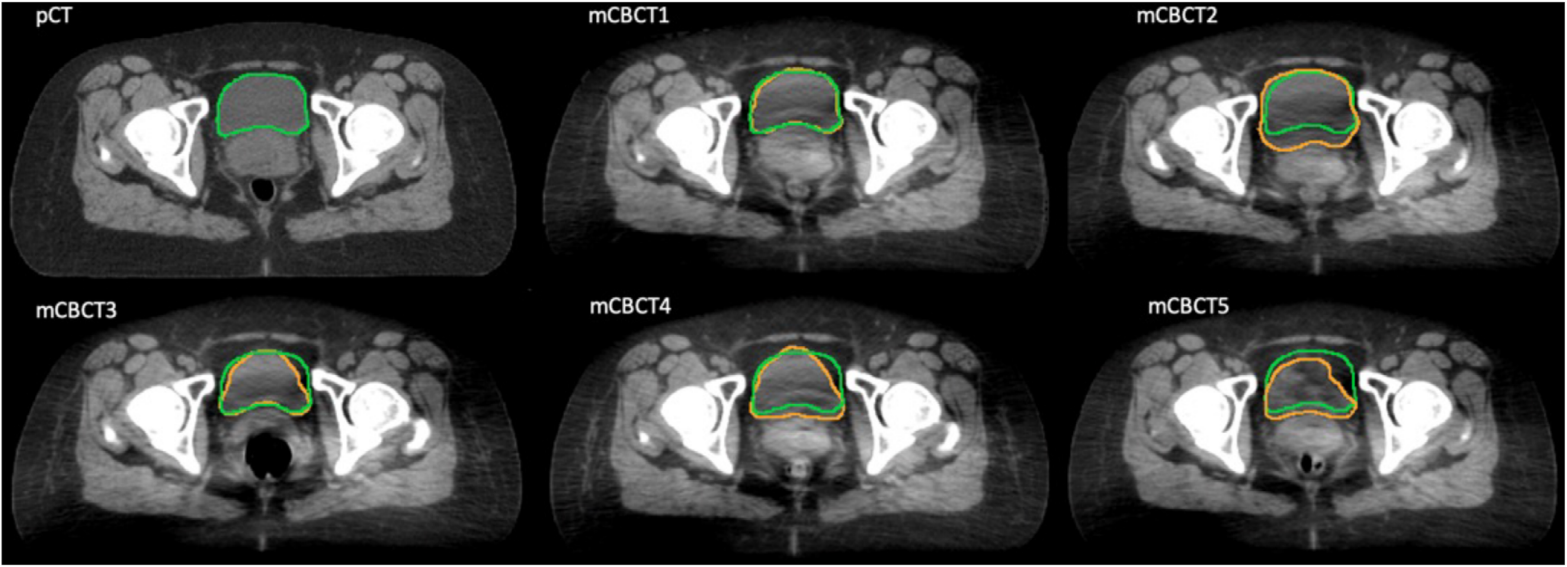
Patient 7 pCT and mCBCT1‐5 display the pCT bladder structure shown in green and each mCBCT‐specific bladder shown in orange. The variation in bladder filling and location between the weekly CBCT compared to the pCT is evident. The larger variation in bladder volumes degraded the DIR‐generated contours and required manual adjustments to be made.

The DSC was used to compare the raw, deformed contours versus the contours created by an expert. Only 25% of the patients had a mean bladder DSC of 0.8 or greater, which was characterized as a good DSC, whereas 50% of the patients had a mean rectum DSC of 0.8 or greater. The bladder experienced a greater variation in location and volume on a weekly basis between the pCT and the 5 CBCTs, which resulted in lower DSC values. Our institution does not use a bladder filling protocol. Studies have indicated that radiation therapy with a full bladder provides better OAR sparing, but unpredictability can still occur due to patient's compliance and anatomical alterations.^25^ Patient 7′s bladder volume at the pCT was 130.65 cc, and throughout the course of treatment, the bladder volume for the mCBCTs ranged from 47.78 to 172.38cc. The DSC across the five mCBCTs ranged from 0.448 to 0.957 for this patient. Figure 14 illustrates the variation in bladder filling and position compared to the pCT bladder structure displayed on each mCBCT at the same slice position.

The rectum typically did not experience large volume changes, and the contour adjustments made were minimal compared to the bladder. The differences between position and volume of the bladder and rectum contributed to the gamma analysis results. If there are variations in the delineation of bladder and rectum volumes between two plans, the calculated dose distribution will be different. Gamma analysis involves comparing dose values at specific points in the calculated and measured dose distributions, and with differences in the contours, the corresponding dose reporting points may be in regions with varying dose levels, thus affecting the comparison and leading to gamma analysis failure.

The additional component of the CBCT image quality also affected the overall results of the DIR causing greater errors similar to Yuan et al.^26^ Previous studies have indicated that CBCT image quality can be impacted by patient size, truncated artifacts, motion artifacts, and imaging parameters. The inherent lower soft tissue contrast in CBCT degrades the registration accuracy and the quality of the deformed contours as a result. The process of manually adjusting the DIR‐generated contours was essential for accurate dosimetric results. Further improvements to the DIR algorithm should be explored, specifically for the female pelvis.

Our results show promise to obtain dosimetric information using the mCBCT. Our study compared the dose from the pCT, dose calculated on the mCBCT in Eclipse using the clinical plan, and dose deformed from the clinical plan onto the mCBCT in MIM. Ninety‐five percent of patients were within 5% of the pD for the bladder D2cc regardless of dose calculation method. For the mean bladder dose, only 75% of patients were within 5%. Patient 6 experienced the largest difference in D2cc between the ecD and pD with 15.3%. Patient 14 experienced a difference of −14.5% between the mdD and pD. In comparison, 85% of patients were within 5% for rectum D2cc and 50% of patients were within 5% for the mean rectum dose. Patient 10 experienced a 12.4% difference between the ecD and pD for D2cc. For all three dose comparisons, patient 20 had percent differences greater than 7% with the largest at −15.1% between the ecD and pD. The lowest percentage differences were between the mdD and pD for bladder D2cc; however, for mean bladder dose, the percentage differences were lower between the ecD and pD. The mdD and ecD tend to perform well against one another for bladder metrics with a negative percentage difference between the two indicating that mdD “underdoses” compared to ecD and pD. For rectum, the mdD and pD had lower percentage differences for D2cc and Dmean. For rectum D2cc, the mdD similarly “underdoses,” but for Dmean, the percentage differences are 50% negative. The PTV45 structure was not deformed but rather transferred onto the mCBCTs. The creation of the PTV accounts for any deformation and patient motion, thus was deemed unnecessary to deform. Regardless of dose method, 95% of patients were within 5% of the pD for D90, and 85% were within 5% for D98. Most patients were within 5% for the evaluated metrics, but for the patients who were outside of this tolerance level suggests that there is a strong indicator for a difference between the delivered dose and the planned dose. Patients in this category could benefit from a replan CT or adapted treatment. The differences in dose typically arose from variations in bladder and rectum filling based on the weekly CBCT image compared to the pCT.

Despite the promising results of calculating dose on mCBCTs for cervical cancer, this study includes limitations. First, the week 1 CBCT image was used as a surrogate for 5 days of treatment. As discussed, the bladder and rectum experienced positional and volumetric variations visible on the weekly CBCT. A better approach would be to use a daily CBCT to calculate the “dose of the day” for cumulative dose. Second, the robustness and nature of the DIR was influenced by factors such as patient size and location of treatment isocenter. The CBCT of a larger patient had significant image quality degradation and truncation. In addition, many GYN patients have involved paraaortic lymph node involvement, which causes the treatment isocenter to be placed superiorly. The CBCT is acquired with the treatment isocenter as the imaging isocenter. For patients with superior isocenters, the organs used for analysis, the bladder and rectum, were not fully contained within the CBCT resulting in patients no longer being eligible for this study.

Dose was calculated using the Hounsfield units (HU) on the CBCT can be treated as a limitation to this study. Giacometti et al. evaluated four techniques for dose calculation on CBCT: (1) applying a standard pCT calibration curve; (2) applying a CBCT site‐specific calibration curve; (3) performing a density override; and (4) using deformable registration.^21^ Method 1 calculates dose on the CBCT images using the pCT calibration curve created for the pCT images using phantom data. Method 2 calculates dose on the CBCT images using a CBCT calibration curve generated from phantom‐specific measurements. Method 3 overrides the CBCT HU values with either HU density or CT numbers from the CT images, and the dose is calculated on the CBCT images. Method 4 deforms the pCT images onto the CBCT images and dose is calculated on the deformed image where the HU values are not modified. A combination of the four methods can also be used for dose calculation on CBCT. Further studies are needed to determine the most accurate method to be used for calculating dose on CBCT.

This study did not perform a transformation to CT HU. However, two methods were used to analyze and compare the dose difference between CBCT HU and CT HU. MIM's SureCalc offers a technique called “Enhanced CBCT.” The process to create the Enhanced CBCT includes the mapping of the CBCT to the pCT intensities and a tool to reduce shading artifacts in the CBCT. The HU values were measured for different materials on the pCT, mCBCT, and Enhanced CBCT using a region of interest (ROI). The HU values and standard deviation were measured for bladder, muscle, fat, cortical bone, and trabecular bone. At our institution, a difference of ±40 HU is acceptable. The results for three patients are shown in Table 7.

**TABLE 7:** Measured HU results for pCT, mCBCT, and Enhanced CBCT for three example patients.

	Bladder	Muscle	Fat	Cortical bone	Trabecular bone
Patient 1					
pCT	11.4 ± 7.0	46.9 ± 7.6	−97.9 ± 15.1	851.0 ± 158.0	143.1 ± 46.6
mCBCT	2.0 ± 8.8	33.8 ± 11.1	−118.1 ± 10.8	818.7 ± 129.7	146.8 ± 42.7
Enhanced	16.3 ± 7.3	26.0 ± 8.3	22.1 ± 10.7	758.1 ± 225.8	4.9 ± 34.3
Patient 3					
pCT	8.4 ± 16.0	54.5 ± 14.8	−95.9 ± 13.6	968.8 ± 128.1	146.0 ± 41.8
mCBCT	3.2 ± 12.5	15.0 ± 9.9	−98.0 ± 11.1	951.2 ± 137.2	128.6 ± 23.4
Enhanced	21.0 ± 7.8	46.8 ± 10.5	28.1 ± 14.8	742.2 ± 102.4	232.3 ± 48.0
Patient 9					
pCT	24.3 ± 13.4	54.2 ± 14.2	−97.4 ± 16.3	666.2 ± 112.0	134.2 ± 98.2
mCBCT	24.2 ± 17.6	33.3 ± 15.9	−96.6 ± 7.6	614.0 ± 164.5	101.7 ± 60.3
Enhanced	41.5 ± 11.6	20.5 ± 14.4	21.1 ± 11.7	405.6 ± 97.0	26.1 ± 80.9

Comparing the pCT and mCBCT for all three patients, patient 9 has a difference of 52 HU for cortical bone. All other differences are within ± 40 HU for pCT and mCBCT. Comparing the pCT and Enhanced CBCT, patient 1 has a fat difference of 120 HU, cortical bone difference of 93 HU, and a trabecular bone difference of 138 HU. For patient 3, the fat difference is 124 HU, the cortical bone difference is 86 HU, and the trabecular bone difference is 86 HU. For patient 9, the fat difference is 118 HU, the cortical bone difference is 260 HU, and the trabecular bone difference is 108 HU. All three patients are within ± 40 HU for bladder and muscle. The larger differences in HU for fat and bone on the Enhanced CBCT is due to the correction step that assumes a single tissue class for the whole body, and using the pCT as a reference, maps the CBCT to pCT intensities. In MIM, this correction step was used for CBCT image quality that is less than the image quality used for this study. The result is over‐correction on the CBCT images resulting in larger HU discrepancies to the pCT. The mCBCT HU is closer to the pCT HU, which supports our decision to calculate dose on the CBCT HU.

**FIGURE 15:**
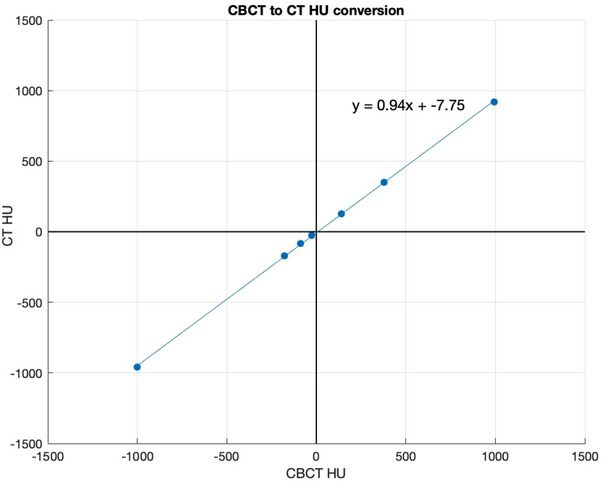
CBCT to CT HU conversion using monthly QA data from the Catphan 600 phantom for our institution's CT and OBI.

Another method involves the data from monthly QA for the OBI CBCT images. Look‐up tables exist at our institution, which provide the HU values for phantom data for the OBI and CT scanner. The Catphan 600 (The Phantom Laboratory, Salem, New York, USA) was used for HU measurements. A linear fit function can be applied to the CBCT HU as shown in Figure 15.

## 5 CONCLUSION

This study presents the use of weekly CBCT images for dose calculation and dose summation for patients with cervical cancer. The performance of a commercial DIR algorithm was evaluated for patients with cervical cancer and two dose calculation and dose summation methods were investigated. Our results indicate that both methods produce similar dose summation results. The proposed workflow should be used on a case‐by‐case basis when the weekly CBCT shows marked volume difference in OAR from the pCT and can be used to understand the actual accumulated dose to OAR compared to the planning dose.

## AUTHOR CONTRIBUTIONS

Carolyn Eckrich: lead author, workflow creation, data acquisition, manuscript revision, and final review. Anna Rodrigues and Oana Craciunescu: conception and design of study, data acquisition, manuscript revision, equal contributions as senior authors. Brandon Lee: workflow creation, manuscript review. Chunhao Wang: data review, manuscript review. Kim Light: lead dosimetrist for treatment plans. Junzo Chino: contour review and adjustment, data review, manuscript review.

## CONFLICT OF INTEREST STATEMENT

The authors declare no conflicts of interest.

## APPENDIX A

### A.1

**TABLE A1:** Bladder DSC values for five weekly mCBCTs.

Bladder	mCBCT1	mCBCT2	mCBCT3	mCBCT4	mCBCT5	Mean ± SD
Patient 1	0.68	0.82	0.79	0.87	0.91	0.82 ± 0.08
Patient 2	0.55	0.45	0.78	0.76	0.74	0.66 ± 0.13
Patient 3	0.86	0.52	0.55	0.50	0.75	0.63 ± 0.14
Patient 4	0.92	0.96	0.88	0.94	0.69	0.88 ± 0.10
Patient 5	0.54	0.47	0.54	0.70	0.6	0.57 ± 0.08
Patient 6	0.81	0.84	0.88	0.63	0.76	0.78 ± 0.18
Patient 7	0.96	0.84	0.70	0.58	0.45	0.70 ± 0.07
Patient 8	0.51	0.50	0.67	0.62	0.61	0.58 ± 0.06
Patient 9	0.78	0.78	0.81	0.73	0.92	0.81 ± 0.06
Patient 10	0.83	0.92	0.62	0.90	0.64	0.78 ± 0.12
Patient 11	0.64	0.90	0.40	0.46	0.81	0.64 ± 0.19
Patient 12	0.80	0.83	0.83	0.73	0.83	0.80 ± 0.04
Patient 13	0.81	0.84	0.74	0.73	0.62	0.74 ± 0.08
Patient 14	0.67	0.51	0.74	0.45	0.81	0.64 ± 0.14
Patient 15	0.42	0.52	0.56	0.58	0.43	0.50 ± 0.06
Patient 16	0.80	0.66	0.63	0.69	0.83	0.72 ± 0.08
Patient 17	0.61	0.57	0.64	0.33	0.54	0.54 ± 0.11
Patient 18	0.56	0.56	0.30	0.40	0.46	0.46 ± 0.10
Patient 19	0.99	0.41	0.48	0.39	0.49	0.55 ± 0.22
Patient 20	0.87	0.83	0.89	0.81	0.83	0.85 ± 0.03

**TABLE A2:** Rectum DSC values for five weekly mCBCTs.

Rectum	mCBCT1	mCBCT2	mCBCT3	mCBCT4	mCBCT5	Mean ± SD
Patient 1	0.82	0.90	0.84	0.86	0.83	0.85 ± 0.03
Patient 2	0.72	0.68	0.74	0.69	0.84	0.73 ± 0.06
Patient 3	0.97	0.96	0.92	0.93	0.85	0.93 ± 0.04
Patient 4	0.85	0.89	0.92	0.86	0.87	0.88 ± 0.03
Patient 5	0.88	0.88	0.82	0.84	0.85	0.85 ± 0.02
Patient 6	0.66	0.68	0.65	0.69	0.61	0.66 ± 0.03
Patient 7	0.90	0.85	0.79	0.86	0.87	0.85 ± 0.04
Patient 8	0.64	0.54	0.65	0.63	0.65	0.62 ± 0.04
Patient 9	0.83	0.77	0.75	0.71	0.70	0.75 ± 0.04
Patient 10	0.85	0.95	0.89	0.95	0.84	0.90 ± 0.05
Patient 11	0.85	0.75	0.69	0.68	0.88	0.77 ± 0.08
Patient 12	0.76	0.62	0.79	0.68	0.82	0.73 ± 0.07
Patient 13	0.79	0.84	0.79	0.80	0.80	0.80 ± 0.02
Patient 14	0.82	0.74	0.71	0.81	0.72	0.76 ± 0.05
Patient 15	0.77	0.82	0.72	0.83	0.78	0.79 ± 0.04
Patient 16	0.91	0.71	0.70	0.80	0.88	0.80 ± 0.08
Patient 17	0.69	0.69	0.73	0.80	0.78	0.74 ± 0.05
Patient 18	0.80	0.85	0.86	0.89	0.82	0.84 ± 0.03
Patient 19	0.88	0.94	0.84	0.80	0.79	0.85 ± 0.06
Patient 20	0.82	0.84	0.79	0.88	0.89	0.84 ± 0.04
